# Vertical Jumping for Legged Robot Based on Quadratic Programming

**DOI:** 10.3390/s21113679

**Published:** 2021-05-25

**Authors:** Dingkui Tian, Junyao Gao, Xuanyang Shi, Yizhou Lu, Chuzhao Liu

**Affiliations:** 1 School of Mechatronical Engineering, Intelligent Robotics Institute, Beijing Institute of Technology, Beijing 100081, China; tiandingkui@bit.edu.cn (D.T.); shixuanyang@bit.edu.cn (X.S.); 3120180169@bit.edu.cn (Y.L.); 3120150091@bit.edu.cn (C.L.); 2 Beijing Advanced Innovation Center for Intelligent Robots and Systems, Beijing 100081, China

**Keywords:** hierarchical quadratic programming, quadratic programming, vertical jumping, full dynamic

## Abstract

The highly dynamic legged jumping motion is a challenging research topic because of the lack of established control schemes that handle over-constrained control objectives well in the stance phase, which are coupled and affect each other, and control robot’s posture in the flight phase, in which the robot is underactuated owing to the foot leaving the ground. This paper introduces an approach of realizing the cyclic vertical jumping motion of a planar simplified legged robot that formulates the jump problem within a quadratic-programming (QP)-based framework. Unlike prior works, which have added different weights in front of control tasks to express the relative hierarchy of tasks, in our framework, the hierarchical quadratic programming (HQP) control strategy is used to guarantee the strict prioritization of the center of mass (CoM) in the stance phase while split dynamic equations are incorporated into the unified quadratic-programming framework to restrict the robot’s posture to be near a desired constant value in the flight phase. The controller is tested in two simulation environments with and without the flight phase controller, the results validate the flight phase controller, with the HQP controller having a maximum error of the CoM in the x direction and y direction of 0.47 and 0.82 cm and thus enabling the strict prioritization of the CoM.

## 1. Introduction

Due to the constraints of underactuation, high dimensionality, and the ground reaction force in legged robots, it is remarkably challenging to control a highly dynamic legged robot’s jumping motion. In the stance phase, the most common solution for jumping is to reduce the full dynamics to a canonical spring-loaded inverted pendulum (SLIP), which renders the control for legged robots computationally tractable and predicts the energy wave and ground reaction force during the jumping motion in the stance phase [1]. A nonlinear controller is employed to synchronize the biped dynamics and SLIP [2,3,4,5,6], which is an effective solution, but makes it difficult to introduce constraints, such as the stability of the robot and acceleration of the joints, into the controller or to guarantee strict prioritization in the overconstrained objective. Additionally, the majority of previous approaches focus on reducing the angular momentum in the center of mass (CoM) at the launch phase [7], and few studies have paid close attention to how to adjust the position and attitude of the robot during the flight phase.

### 1.1. Related Work—Hierarchical Task Controllers

In overconstrained tasks for robots, controlling the hierarchies of the tasks is very important. There are two main solutions to solving overconstrained problems: adding weights to the control tasks and hierarchical task control.

In dynamic stair climbing, Caron et al. extended walking stabilization using linear inverted pendulum tracking with a QP-based wrench distribution and a whole-body admittance controller [8]. Ott et al. presented a new balance control approach for regulating the CoM position and trunk orientation of a bipedal robot in a compliant way in Ott et al. [9], Feng et al. [10] described the method of full body humanoid control developed for the DARPA in Feng et al. [10], and Lee and Goswami presented an approach for maintaining the balance of humanoid robots using control momentum in Lee and Goswami [11]. Jiang et al. [12] showed the possibility for a bipedal robot driven by electric motors to perform a high jump. Ahn et al. [13,14] described a method of generating an optimal periodic hopping trajectory for legged robots and verified its performance in experiments. Wan et al. [15] investigated an optimal jumping motion of a four-link legged robot that minimizes the necessary joint torques during the motion. Nguyen et al. [16] presented a novel methodology for implementing optimized jumping behavior on quadruped robots, which included efficient trajectory optimization, a precise high-frequency tracking controller and a robust landing controller for stabilizing the robot body position and orientation after impact. Ding and Park [17] introduced a novel method for actuator design that exploits the electromagnetic motors’ torque and speed potential in jumping applications. Different weights have been applied to different control objectives to reflect priority [8,9,10,11,12,13,14,15,16,17]. These approaches are intuitive and easy to implement on a digital computer; however, they only express relative priority and it is difficult to guarantee strict prioritization.

Hierarchical methods are based on pseudo-inverse techniques [18,19], but the iterative null-space projection in Kim et al. [20] and Hutter et al. [21] is limited because inequality constraints, such as joint acceleration limits or friction cone constraints, cannot be properly handled. Instead of simplifying the robot model to decrease the computational overhead, Herzog et al. [22,23] combined the approaches in Nguyen et al. [16] and Ding et al. [17] to introduce a simplified method for real-time control using a reformulation of existing algorithms in [24,25].

### 1.2. Related Work—Controllers in the Flight Phase

As the robot’s foot leaves the ground in the flight phase, keeping the position and attitude of the robot near the desired values is challenging.

Barkan et al. [4,7] and Justin et al. [5] maintained the angular momentum with respect to the CoM of the robot to track the desired value, ensuring that the angular momentum of the robot is small enough before the robot’s foot leaves the ground, so that the leg only needs to be swung to prepare for landing.

Aoustin and Formalskii in [26,27] introduced vertical upward jumping for a planar biped with and without a foot, respectively. Xiong and Ames presented a design for controlling hopping in a three-dimensional (3-D) legged robot Cassie, whose leg models included spring stiffness and damping to control hopping behaviors [28]. Tamaddoni et al. introduced a nonlinear controller to synchronize the biped dynamics and spring-mass dynamics to perform a stable locomotion corresponding to the SLIP model [29]. Vermeulen et al. presented a real-time control algorithm for a one-legged planar hopping robot, which was able to hop on irregular terrain by changing its objective locomotion parameters [2]. Scott et al. [30] described a collection of optimization algorithms for achieving the dynamic planning, control, and state estimation of a bipedal robot designed to operate reliably in complex environments. Miyadaira et al. [31] proposed two approaches of analyzing the movement of a robot with an articulated leg (without a toe) having three degrees of freedom. Schlossman et al. [32] formulated the trajectory optimization problem for a series of elastic robots in a novel way adopting sequential linear programming. Zhang et al. [33] presented the design of a jumping robot inspired by the jumping locomotion of locusts. All these studies focused only on how the robot realizes jumping, little attention was paid to the change in angular momentum in the launch phase and the control of the robot during the flight phase.

Nunez et al. introduced an approach based on a single mass point model to enable humanoid robots to jump vertically. A sliding mode controller is used to ensure the robot’s CoM tracks the desired value over the whole jump, including the flight phase [34,35].

### 1.3. Proposed Approach and Contribution

**Figure 1:**
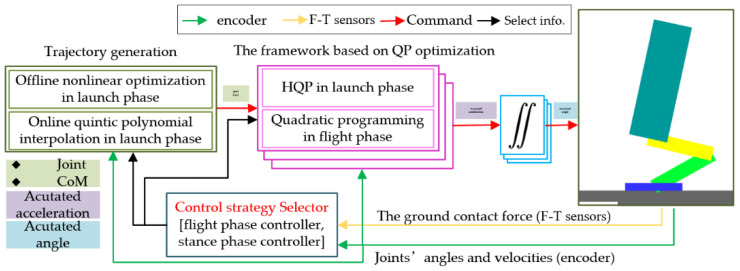
Overview of the online control algorithm.

In this paper, we propose an online unified framework based on QP. Our scheme consists of trajectory generation and an online unified framework, as shown in Figure 1. Moreover, the trajectory generation in the launch phase is converted into an offline nonlinear optimization problem and the trajectories of the actuated joints and CoM are described by online quintic polynomial interpolation. The control problems can be successfully unified into a framework based on QP optimization in real time.

The main contributions of this paper are as follows:

(1) The launch problem for a robot is essentially to maintain the CoM velocity and acceleration enough to overcome gravity, so tracking the desired CoM trajectory is the highest priority. To strictly prioritize the CoM trajectory and decrease the computational overhead, we assume that the foot stays in contact with the ground to reduce the degrees of freedom of the robot and simplify the dynamic equations. Moreover, HQP control strategy is used instead of assigning multiplicative factors (i.e., weights) to the control objectives to represent different priorities.
(2) In the flight phase, the dynamic equation is split into the actuated part and the underactuated part. Based on the reformed dynamic equation, the overconstrained control problem is incorporated into the online unified QP framework to keep the robot’s posture near a desired constant.

## 2. Planar Biped and its Dynamical Model

In this section, the mathematical modeling for the biped jump is introduced, which was used in the simulation and relies upon the different phases of the biped jump.

### 2.1. Biped

**Figure 2:**
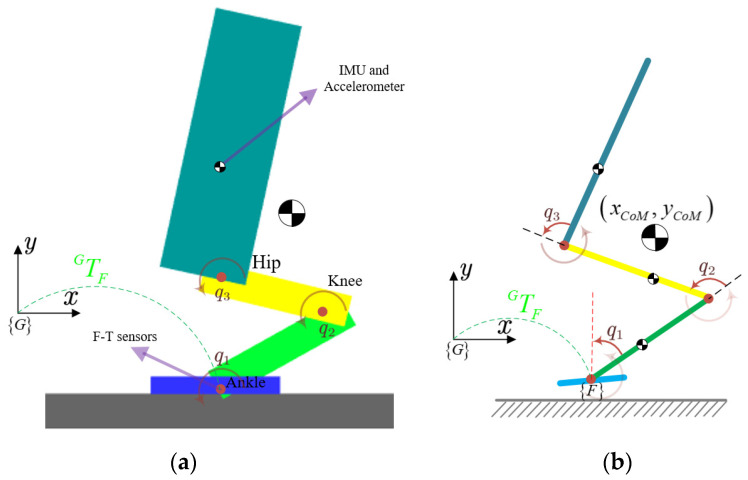
Jumping models for the planar biped robot in simulations and its generalized coordinates. (**a**) Jumping models in simulation. (**b**) Simplified configuration of the mechanical structure. {G} is attached to the ground. The robot’s foot frame is denoted {F}, and homogeneous transformation matrix TFG converts coordinates from {G} to {F}.

The generalized coordinates for robot jumping motion are depicted in the diagram of a planar single-legged three-link jumping robot shown in Figure 2. To simplify the research problem, we make two assumptions:

(1) The robot is generally arranged symmetrically in the sagittal plane.
(2) Only the hip, knee, and ankle joints of the robot can move. The other joints, such as the shoulder and elbow joints, are fixed.

Hence, the jumping of the robot is constrained in the *x − y* plane and characterized by a single-legged three-link model.

As shown in Figure 2, to control the full dynamical model, we introduce a floating frame {F} in the jumping model, which is fixed to the intersection of the foot and shank of the robot. We attached an inertial measurement unit (IMU) and accelerometer to the CoM of the torso of the robot, and they are respectively used to measure the robot’s attitude and position relative to the inertial frame system {G}. A six-axis universal force/torque (F-T) is implemented between the foot and the ankle to detect the forces and torques applied to the robot’s foot.

The torso, thigh, shank, and foot are connected to each other in sequence. The mass of a human foot is much less than the mass of the thigh, shank, and torso, so we treat the foot in this model as massless. However, the case of a massless foot is not equivalent to the case of a point foot. Variables $q_{1}$, $q_{2}$ and $q_{3}$ are the angles of the ankle, knee, and hip, respectively, and the positive direction is indicated in Figure 2 by a brown arrow. Moreover, x and y are used to denote the horizontal and vertical positions, respectively, and $q_{f}$ denotes the angle from the foot frame {F} with respect to the inertial frame {G}.

Lengths $L_{1}$, $L_{2}$ and $L_{3}$ denote the length of the shank, thigh, and torso links, respectively, and the distance from the CoM of each link of the robot to the proximal joint frame is denoted as $L_{ci}$. We denote by $m_{i}$ the masses of these links and $I_{ci}$ the inertial moment of these links. The total mass of the biped is expressed by $m_{t}$. The torques acting on the ankle, knee, and hip joints are denoted by $\tau_{1}$, $\tau_{2}$ and $\tau_{3}$, respectively. The inertial parameters of the robot used in the simulation are listed in Table 1.

### 2.2. Planar Dynamic Model

According to whether the foot is in contact with the ground and a generalized force from the ground acts on the foot, we divide the dynamics model of the robot into a ground phase and a flight phase.

Let us introduce vectors $q_{c}={\left[x,y\right]}^{T}$ and $q_{j}={\left[q_{f},q_{1},q_{2},q_{3}\right]}^{T}$ (*T* denotes transposition). We define $q={\left[q_{c},q_{j}\right]}^{T}$. As only the hip, knee, and ankle joints are actuated joints and the others are underactuated joints, we define $\tau ={\left[\tau_{1},\tau_{2},\tau_{3}\right]}^{T}$. Using the Newton–Euler method, the dynamic equation for a jumping motion can be obtained in the following compact matrix form:

(1) $$M\left(q\right)\ddot{q}+V\left(q,\dot{q}\right)+G\left(q\right)=B\tau +J^{T}F_{ext}$$

where M(q) is the inertial matrix; $V\left(q,\dot{q}\right)+G\left(q\right)$ is the sum of gravitational, centrifugal, and Coriolis forces; B is the torque selection matrix; $F_{ext}$ is the external force; J is the corresponding Jacobian matrix.

### 2.3. Time Sequence of Vertical Jumping

**Figure 3:**
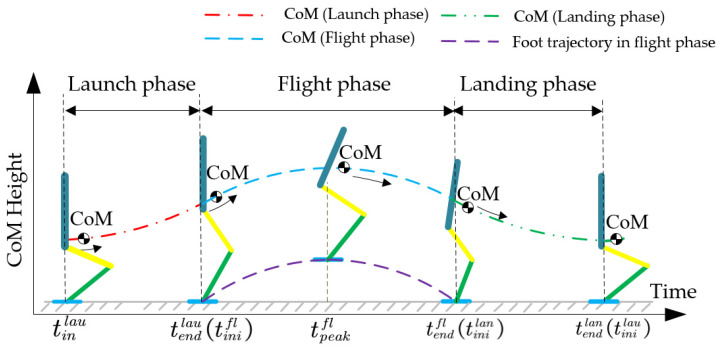
Time sequence of the vertical jumping motion.

As shown in Figure 3, the whole jumping process of the robot can be simply divided into two parts: the stance phase, when the robot foot is in contact with the ground, which provides thrust against gravity, and the flight phase, when the foot leaves the ground and there is no interaction between them. The stance phase consists of two subphases: the launch phase when the robot starts to jump but its foot is still in contact with the ground and the landing phase, when the foot contacts the ground again after the flight phase and the robot prepares for the next jump.

### 2.4. Simplification of the Full Dynamics

A hierarchical full dynamic controller based on HQP is a very promising approach for controlling the strict hierarchies of tasks, however, HQP substantially increases the computational overhead of online calculation, which may prevent the controller from operating in real time, so it is necessary to decrease the computational overhead. Reducing the number of variables is a straightforward and sufficiently effective method. The full dynamics in equation should thus be simplified to reduce the size of the problem.

#### 2.4.1. Simplification in the Stance Phase

**Figure 4:**
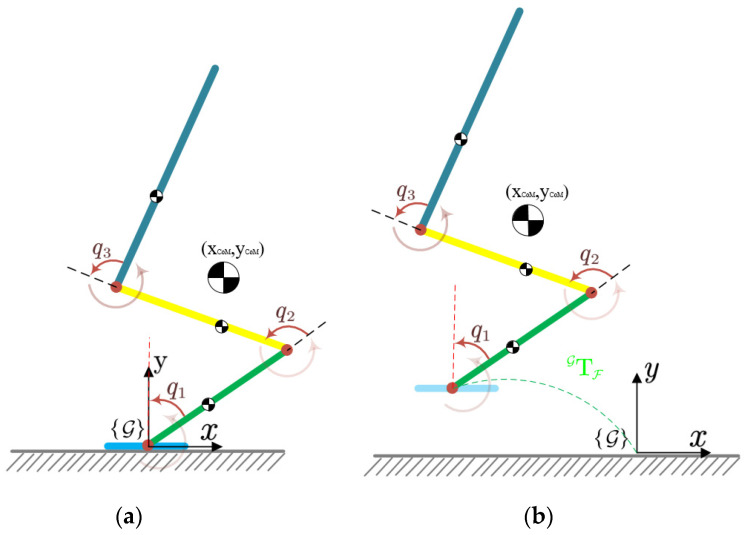
Dynamic model (**a**) in the stance phase and (**b**) in the flight phase.

To simplify the jumping problem during the stance phase, we assume that the robot’s foot remains in contact with the ground; no sliding or rotation between the foot and the ground is allowed. As shown in Figure 4a, the full dynamic model for the jump problem reduces to a degenerative dynamic model, which consists of only three actuated joints, the hip, knee, and ankle joints, and we define joint vectors $q_{s}={\left[q_{1},q_{2},q_{3}\right]}^{T}$ and actuated torques $\tau_{s}\in {\mathbb{R}}^{n_{s}}$. The motion of the robot in the stance phase, with the foot on the ground, is governed by

(2) $$M\left(q_{s}\right){\ddot{q}}_{s}+V\left(q_{s},{\dot{q}}_{s}\right)+G\left(q_{s}\right)=\tau_{s}$$

#### 2.4.2. Simplification in the Flight Phase

As depicted in Figure 4b, in the flight phase, because the massless foot of the robot leaves the ground and no external force from the ground is able to act on the foot, the ankle joint degenerates to an underactuated joint. The full dynamic model reduces to a degenerative dynamic model that consists of five joints and two actuated joints, i.e., the knee and hip joints. We define vector $q_{fl}={\left[x,y,q_{1},q_{2},q_{3}\right]}^{T}$ and actuated torques $\tau_{fl}={\left[\tau_{2},\tau_{3}\right]}^{T}$. The movement of the robot in the flight phase is characterized by

(3) $$M\left(q_{fl}\right){\ddot{q}}_{fl}+V\left(q_{fl},{\dot{q}}_{fl}\right)+G\left(q_{fl}\right)=B_{fl}\tau_{fl}$$

## 3. Trajectory Generation

Trajectory generation for vertical jumping consists of two parts: offline optimization in the stance phase and online interpolation in the landing phase. From the degenerative dynamic equation of the robot in the stance phase, we know that input torques τ only depend on the actuated joint information. Therefore, the problem of searching for the desired reference trajectory is transformed into a problem of seeking the Bèzier polynomial parameters through an offline nonlinear optimization. To generate the trajectory for the landing phase, the joint information of the initial landing phase can be obtained by online sensors and using the initial position, velocity, and acceleration of the actuated joints in the launch phase as the terminal values of the landing phase. Hence, quintic polynomial interpolation is employed to describe the trajectories of the actuated joints, and the CoM trajectory can be calculated from the known joint information.

### 3.1. Trajectory Optimization in the Launch Phase

#### 3.1.1. Transformation of Trajectory Generation in the Launch Phase

From Equation (2), torques τ depend on $q_{s}$, ${\dot{q}}_{s}$ and ${\ddot{q}}_{s}$. In other words, τ can be represented in terms of function $q_{s}\left(t\right)$. An optimization procedure for seeking the actuated joint trajectories is proposed to determine feasible solutions for the parameters of three smooth 15th-order Bèzier polynomials, which are used to represent actuated joint angles. A typical Bèzier polynomial θ(t) is in the form of

(4) $$\theta \left(t\right)=\sum_{i=0}^{m}\frac{m!}{i!\left(m-i\right)!}t^{i}{\left(1-\frac{t}{t_{0}}\right)}^{m-i}\cdot x_{i}$$

where *m* is the order of the polynomial, $t_{0}$ is the time of the Bèzier polynomial, and $x_{i}$ is the control point. The Bèzier polynomials of the actuated joints can generally be parameterized as

(5) $$\begin{array}{cc} q_{s}^{i}=H_{i}L\left(t\right) & \left(i=1,2,3\right) \end{array}$$

where $q_{s}^{i}$ is the trajectory function for the *i*-th actuated joint, $H_{i}$ is the control point matrix of the associated Bèzier polynomial, L(t) is constituted by the t corresponding to $H_{i}$. Therefore, the problem of searching for the optimal trajectories of the actuated joints and CoM in the stance phase becomes a problem of seeking feasible parameters $H_{i}$ for the Bèzier polynomials.

#### 3.1.2. Cost Function of Nonlinear Optimization

In a DC motor, torque is proportional to the actuated current of the motor, and electric power is proportional to the square of the current. Due to the limited actuation capacities of the joints in an actual robot, it is prudent to minimize the actuated energy and joint accelerations while limiting the upper and lower bounds of the joint torques and accelerations. The torques and accelerations are penalized as follows:

(6) $$J_{\tau q}=\sum_{k=1}^{N}{\left\|\tau_{s\_k}\right\|}_{w_{\tau }}+{\left\|{\ddot{q}}_{s\_k}\right\|}_{w_{q}}$$

where, $\tau_{s\_k}$ and ${\ddot{q}}_{s\_k}$ are the joint torque and acceleration at time step *k*, respectively. Furthermore, $w_{\tau }$ and $w_{q}$ are matrices of weights for $\tau_{s\_k}$ and ${\ddot{q}}_{s\_k}$, respectively.

To avoid high frequency oscillation, we penalize changes in τ and $\ddot{q}$ with

(7) $$J_{ch}=\sum_{k=1}^{N}{\left\|\tau_{s\_k}-\tau_{s\_k-1}\right\|}_{w_{\tau ch}}+{\left\|{\ddot{q}}_{s\_k}-{\ddot{q}}_{s\_k-1}\right\|}_{w_{qch}}$$

where, $w_{\tau ch}$ and $w_{qch}$ are the weight matrices for the change in torques and accelerations, respectively.

Therefore, the cost function of the trajectory optimization problem in the launch phase is formulated as follows:

(8) $$J=\sum_{k=1}^{N}\left[{\left\|\tau_{s\_k}\right\|}_{w_{\tau }}+{\left\|{\ddot{q}}_{s\_k}\right\|}_{w_{q}}+{\left\|\tau_{s\_k}-\tau_{s\_k-1}\right\|}_{w_{\tau ch}}++{\left\|{\ddot{q}}_{s\_k}-{\ddot{q}}_{s\_k-1}\right\|}_{w_{qch}}\right]$$

#### 3.1.3. Constraints for Achieving a Stable Launch

(1) Limits of the joint angles, velocities, and accelerations

In practical robot applications, joint angles, velocities, and accelerations must remain within their physical ranges and cannot exceed their bounds; otherwise, it is easy to damage a joint’s mechanical configuration. Hence, the physical ranges of the joints are represented as follows:

(9) $$\left\{ \begin{array}{l} \underline{q}\leq q_{s\_k}\leq \bar{q}\\ \dot{\underline{q}}\leq {\dot{q}}_{s\_k}\leq \bar{\dot{q}}\\ \ddot{\underline{q}}\leq {\ddot{q}}_{s\_k}\leq \bar{\ddot{q}} \end{array}\right.$$

where $\underline{q}$ and $\bar{q}$ are the upper and lower boundary matrices for the angles; $\dot{\underline{q}}$ and $\bar{\dot{q}}$ are the upper and lower boundary matrices for the velocities; $\ddot{\underline{q}}$ and $\bar{\ddot{q}}$ are the upper and lower boundary matrices for the accelerations, respectively.

(2) Limits of the joint torques

With regard to the capacity of the motor and gearbox, it is important to limit the output torques of the robot’s joints; therefore, the following torque constraints are imposed:

(10) $$\underline{\tau }\leq \tau_{s\_k}\leq \bar{\tau }$$

where, $\underline{\tau }$ and $\bar{\tau }$ are the upper and lower boundaries of the torques.

(3) Constraints of the CoM trajectory

As this study focuses on vertical jumps, the horizontal component of the CoM trajectory is always zero, which is formulated as follows:

(11) $$\left\{ \begin{array}{l} x_{CoM}=0\\ {\dot{x}}_{CoM}=0\\ {\ddot{x}}_{CoM}=0 \end{array}\right.$$

where, $x_{CoM}$, ${\dot{x}}_{CoM}$ and ${\ddot{x}}_{CoM}$, respectively, denote the position, velocity, and acceleration of the horizontal CoM trajectory component.

The robot should continuously expand its body to increase the CoM position in the launch phase. Therefore, the vertical position of the CoM at each time step should be higher than the previous one, as follows:

(12) $$y_{CoM\_s\_k-1}\leq y_{CoM\_s\_k}$$

where $y_{CoM\_s\_k-1}$ and $y_{CoM\_s\_k}$ denote the vertical position of the CoM at time steps *k* − 1 and *k*, respectively.

(4) Ground reaction force constraints

In the launch phase, the vertical force $f_{s\_y}$ acting on the robot is always vertical with respect to the ground, and the horizontal force $f_{s\_x}$ is always opposite to the direction of motion and parallel with the ground, as follows:

(13) $$\left\{ \begin{array}{l} f_{s\_x}=m_{t}{\ddot{x}}_{CoM}=0\\ f_{s\_y}=m_{t}\left({\ddot{y}}_{CoM}+g\right) \end{array}\right.$$

where ${\ddot{y}}_{CoM}$ is acceleration of the CoM in the vertical component. Equation (11) results in $f_{s\_x}=0$. Based on the physical meaning of the foot in contact with the ground and Equation (13), we can conclude that $f_{s\_y}$ is non-negative.

Sliding is assumed not to occur between the foot and the ground, so the friction cone constraint can be formed as follows:

(14) $$\left\|f_{s\_x}\right\|\leq u_{s}f_{s\_y}$$

where $u_{s}$ is the coefficient of friction. The CoM position is zero in the horizontal direction, which means that Equation (14) always holds.

To protect the robot’s mechanical structure, the ground reaction force applied to the robot cannot exceed the maximum force $f_{\max}$.

(15) $$\sqrt{f_{x}^{2}+f_{y}^{2}}\leq f_{\max}\to f_{y}^{}\leq f_{\max}$$

(5) Initial body configuration

The initial posture of the robot is defined by

(16) $$\begin{array}{cc} \left\{ \begin{array}{l} q_{s\_i}=q_{s\_i,d}\\ {\dot{q}}_{s\_i}={\dot{q}}_{s\_i,d}\\ {\ddot{q}}_{s\_i}={\ddot{q}}_{s\_i,d} \end{array}\right. & i=0,N \end{array}$$

where $q_{s\_i,d}$, ${\dot{q}}_{s\_i,d}$ and ${\ddot{q}}_{s\_i,d}$ are given joint angle, velocity, and acceleration vectors at time step 0 and N (the launch moment), respectively.

(6) Stability constraints

To ensure that the foot will not tip over and cause the robot to fall down, the zero moment point (ZMP) should be kept inside the support polygon of the foot, which can be expressed as follows:

(17) $$L_{\min}\leq ZMP_{x}\leq L_{\max}$$

where $L_{\min}$ and $L_{\max}$ are the minimum and maximum values of the foot in the horizontal direction.

Finally, the optimization problem can be cast as

(18) $$\underset{H}{\min}\begin{array}{c} \left(\sum_{k=1}^{N}\left[{\left\|\tau_{s\_k}\right\|}_{w_{\tau }}+{\left\|{\ddot{q}}_{s\_k}\right\|}_{w_{q}}+{\left\|\tau_{s\_k}-\tau_{s\_k-1}\right\|}_{w_{\tau ch}}++{\left\|{\ddot{q}}_{s\_k}-{\ddot{q}}_{s\_k-1}\right\|}_{w_{qch}}\right]\right)\\ \;s.t.\;\mathrm{Equations}\;(9)\sim (13)\;\mathrm{and}\;(15)\sim (17) \end{array}$$

where H is vector constituted by the control points of the associated polynomial. s.t. represent *subject to*.

**Figure 5:**
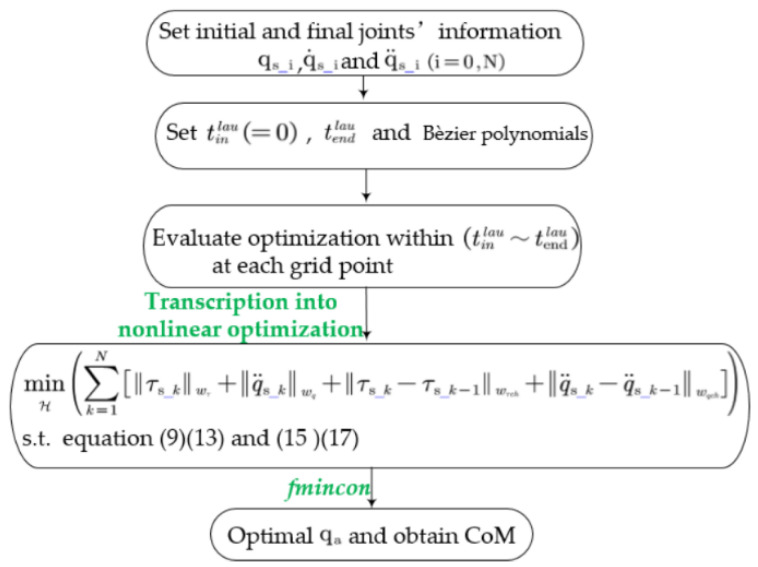
Optimization procedure.

As shown in Figure 5, we first specify the initial joint information (angles, velocities, and accelerations) according to the initial configuration of the robot. The terminal joint information is obtained from the configuration and the launch velocity of the CoM. Next, we set the duration of the launch phase to 0.2 s according to the physiological laws of humans and animals as well as the research results of robots in [36,37,38,39], and describe the joints using Bèzier polynomials. Next, the joints are discretized at each sampling time. Finally, the problem of generating the trajectory in the launch phase is converted into a nonlinear optimization problem and the standard discrete nonlinear optimization problem is solved numerically by the function *fmincon* in the MATLAB Optimization Toolbox.

The weights and parameters of the nonlinear optimization for the launch phase are listed in Table 2. They are identical to the geometric dimensions and the inertial properties of the robot in the simulation.

**Figure 6:**
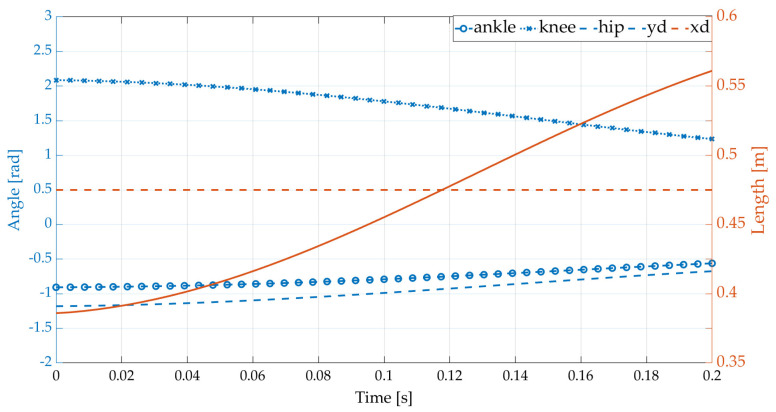
Trajectory of the actuated joints and CoM in the vertical component.

As shown in Figure 6, the actuated joints extend to push the CoM until the robot’s vertical component of acceleration is completely capable of overcoming gravity under various constraints, which is intuitively satisfactory for achieving human-like launch motion.

### 3.2. Trajectory Generation in the Landing Phase

The robot’s joint information at the moment of landing cannot be obtained in advance but can be achieved by online sensors. Therefore, we are only able to employ online interpolation to characterize the trajectories in the landing phase. To achieve periodic jumping, the robot at the terminal moment in the landing phase should return to the initial configuration in the launch phase, so we select the initial position, velocity, and acceleration of the actuated joints and CoM in the launch phase as the information for the terminal moment of the landing phase. In other words, the initial and final information is known, so we choose quintic polynomial interpolation to describe the trajectories of the actuated joints, and the CoM’s trajectory can be calculated from the quintic polynomial interpolation of the joints.

## 4. Control Algorithm

The algorithm for the vertical jumping motion is composed of two parts, the stance phase and the flight phase, as determined by the ground reaction force. In the stance phase, the degenerative model is adopted to substantially decrease the computational overhead to achieve HQP control. In the flight phase, the dynamic model with a massless foot is employed to keep the robot near the desired posture to prepare for landing.

### 4.1. Control in the Landing Phase

The essence of the jumping problem is to push the mass of the robot to a desired height and velocity so that the robot can fly in the air and realize the desired kinematic performance of an inverted pendulum.

#### 4.1.1. Problem Formulation

(1) Cost function formulation

The acceleration of the CoM in the horizontal and vertical components can be expressed as follows:

(19) $$\left\{ \begin{array}{l} {\ddot{x}}_{CoM}={\dot{J}}_{x}{\dot{q}}_{s}+J_{x}{\ddot{q}}_{s}\\ {\ddot{y}}_{CoM}={\dot{J}}_{y}{\dot{q}}_{s}+J_{y}{\ddot{q}}_{s} \end{array}\right.$$

where $J_{x}$ and $J_{y}$ are the Jacobian vectors of the CoM in the horizontal and vertical components, respectively.

So that the CoM follows the desired trajectory, the input is designed as follows:

(20) $$\left\{ \begin{array}{l} {\ddot{x}}_{CoM}^{in}=k_{px}(x_{CoM}^{d}-x_{CoM}^{})+k_{dx}({\dot{x}}_{CoM}^{d}-{\dot{x}}_{CoM}^{})\\ {\ddot{y}}_{CoM}^{in}=k_{py}(y_{CoM}^{d}-y_{CoM}^{})+k_{dy}({\dot{y}}_{CoM}^{d}-{\dot{y}}_{CoM}^{}) \end{array}\right.$$

where $x_{CoM}^{d}$ and ${\dot{x}}_{CoM}^{d}$ are, respectively, the desired position and velocity of the CoM in the horizontal direction; $y_{CoM}^{d}$ and ${\dot{y}}_{CoM}^{d}$ are, respectively, the desired position and velocity of the CoM in the vertical direction.

The joints are expected to track the planned joint trajectory. Input ${\ddot{q}}_{s}^{in}$ can be calculated as follows:

(21) $${\ddot{q}}_{s}^{in}=k_{pq}(q_{s}^{d}-q_{s}^{})+k_{dq}({\dot{q}}_{s}^{d}-{\dot{q}}_{s}^{})$$

While tracking the desired CoM and actuated joint trajectories, changes in the joint accelerations should be penalized to avoid high frequency oscillation in the actuated joints.

(2) Constraint formulation

Assuming that the foot does not rotate in the stance phase, the ZMP should remain inside the support polygon. Following the argument in [40], the ZMP can be formulated as follows:

(22) $$ZMP_{x}=\frac{x_{CoM}g+x_{CoM}{\ddot{y}}_{CoM}-{\ddot{x}}_{CoM}y_{CoM}}{{\ddot{y}}_{CoM}+g}$$

where the front and length of foot are $L_{\min}$ and $L_{\max}$, respectively, so $L_{\min}\leq ZMP_{x}\leq L_{\max}$ is guaranteed.

To avoid slippage, the friction cone should be approximated by

(23) $$\left\|{\ddot{x}}_{CoM}\right\|\leq u_{s}\left({\ddot{y}}_{CoM}+g\right)$$

where $u_{s}$ is the friction coefficient.

With regard to the capacity of the motor and gearbox, the output torques and accelerations of the joints should be added to the constraints as

(24) $$\ddot{\underline{q}}\leq {\ddot{q}}_{s}\leq \bar{\ddot{q}}$$

At every control cycle, control objective and constraints can be expressed as a linear combination of ${\ddot{q}}_{s}$, which are the optimization variables of our problem. Tasks with the same priorities can be stacked into the following form:

(25) $$\left\{ \begin{array}{l} Ax+a\leq 0\\ Bx+b=0 \end{array}\right.$$

where input $x={\ddot{q}}_{s}$, $A\in {\mathbb{R}}^{(2+2n_{s})\times n_{s}}$, $a\in {\mathbb{R}}^{\left(2+2n_{s}\right)}$, $B\in {\mathbb{R}}^{(2+2n_{s})\times n_{s}}$ and $b\in {\mathbb{R}}^{\left(2+2n_{s}\right)}$, where $n_{s}$ is the number of actuated joints in the robot.

#### 4.1.2. HQP Solver

The jumping control problem in (25) does not have a common solution because it has so many objective and constraints that trade-off against each other. For example, the objective that the CoM should track the desired trajectory might conflict with the objectives of the joints. The final jumping control can be expressed as a QP optimization problem as follows:

(26) $$\begin{array}{l} \underset{x}{\min}\left\|Bx+b\right\|+\left\|\varepsilon \right\|\\ \begin{array}{cc} s.t. & Ax+a\leq \varepsilon \end{array} \end{array}$$

where ε is the regularization term, and B and b are divided into ${\left[B_{1},B_{2}\right]}^{T}$ and ${\left[b_{1},b_{2}\right]}^{T}$ according to priority.

As the control objectives can be in a trade-off relationship with each other, as noted in (26), strict priority cannot be guaranteed. It is common to add different weights to the control objectives to reflect distinct priorities, which does not guarantee strict prioritization. Therefore, HQP is used in the proposed method. Given one solution $x_{r}^{*}$ with priority *r*, the solution for all remaining lower priority $x_{r+1}^{*}$ can be expressed as follows:

(27) $$\left\{ \begin{array}{l} x_{r+1}^{*}=x_{r}^{*}+Z_{r+1}u_{r+1}\\ {\left[B_{1},B_{2},\cdots ,B_{r}\right]}^{T}Z_{r+1}=0 \end{array}\right.$$

The optimization at priority *r* + 1 can be expressed as

(28) $$\begin{array}{l} \min\left\|B_{r+1}x_{r+1}^{*}+b_{r+1}\right\|+\left\|\varepsilon \right\|\\ \begin{array}{cc} s.t. & Ax_{r+1}^{*} \end{array}+a\leq \varepsilon \end{array}$$

Finally, the optimization problem of jumping in Equation (26) is solved in order from high priority to low priority. In essence, the solution for objectives with low priority is searched for in the null space of the high priority solutions.

In Table 3, $I\in {\mathbb{R}}^{n_{s}\times n_{s}}$ is the identity matrix and ${\ddot{q}}_{s}^{N}$ is the output joint’s acceleration in the last time step.

### 4.2. Control in the Flight Phase

During the flight phase, the robot possesses five degrees of freedom, three of which are underactuated and two of which are actuated, i.e., the knee and hip joints. Therefore, the dynamics equation in Equation (3) can be split into two parts, which can be expressed as follows:

(29) $$\left\{ \begin{array}{l} M_{11}{\ddot{q}}_{u\_fl}+M_{12}{\ddot{q}}_{a\_fl}+V_{1}+G_{1}=0\\ M_{21}{\ddot{q}}_{u\_fl}+M_{22}{\ddot{q}}_{a\_fl}+V_{2}+G_{2}=\tau_{fl} \end{array}\right.$$

in which $M_{11}\in {\mathbb{R}}^{3\times 3}$, $M_{12}\in {\mathbb{R}}^{3\times 2}$, $M_{21}\in {\mathbb{R}}^{2\times 3}$ and $M_{22}\in {\mathbb{R}}^{2\times 2}$ constitute the matrix M. Moreover, $V_{1}\in {\mathbb{R}}^{3\times 1}$ and $V_{2}\in {\mathbb{R}}^{2\times 1}$ constitute vector V, and $G_{1}\in {\mathbb{R}}^{3\times 1}$ and $G_{2}\in {\mathbb{R}}^{2\times 1}$ constitute vector G. ${\ddot{q}}_{u\_fl}={\left[x,y,q_{1}\right]}^{T}$ and ${\ddot{q}}_{a\_fl}={\left[q_{2},q_{3}\right]}^{T}$ are, respectively, the underactuated joints and actuated joints.

Hence, because matrix $M_{11}$ is full rank, the accelerations of the underactuated joints can be formulated as follows:

(30) $${\ddot{q}}_{u\_fl}=-M_{11}^{-1}M_{12}{\ddot{q}}_{a\_fl}-M_{11}^{-1}\left(V_{1}+G_{1}\right)$$

To avoid allowing the robot’s joints to deviate far from the desired value and constrain the robot to approximate the desired attitude in the flight phase, the robot’s attitude is controlled. Hence, the input of underactuated joints can be computed by

(31) $${\ddot{q}}_{u\_fl}^{in}=k_{pu}\left(q_{u\_fl}^{d}-q_{u\_fl}^{}\right)+k_{du}\left({\dot{q}}_{u\_fl}^{d}-{\dot{q}}_{u\_fl}^{}\right)$$

where $k_{pu}\in {\mathbb{R}}^{3\times 3}$ and $k_{du}\in {\mathbb{R}}^{3\times 3}$ are diagonal matrices.

The actuated joint inputs can be computed by

(32) $${\ddot{q}}_{a\_fl}^{in}=k_{pa}\left(q_{a\_fl}^{d}-q_{a\_fl}^{}\right)+k_{da}\left({\dot{q}}_{a\_fl}^{d}-{\dot{q}}_{a\_fl}^{}\right)$$

where $k_{pa}\in {\mathbb{R}}^{2\times 2}$ and $k_{da}\in {\mathbb{R}}^{2\times 2}$ are diagonal matrices.

The control objective is to restrict the robot’s attitude so that it is close to the desired value, not to track the desired trajectories to prepare for landing, so strict prioritization does not need to be guaranteed. Therefore, instead of using HQP, we added different weights to different control targets to describe the priority. Hence, the control for the robot in the flight phase can be formulated as a QP optimization problem. The unified QP optimization problem can be cast as follows:

(33) $$\begin{array}{l} \underset{x}{\min}{\left\|Bx+b\right\|}_{w}+\left\|\varepsilon \right\|\\ \begin{array}{cc} s.t. & Ax+a\leq \varepsilon \end{array} \end{array}$$

where $A=\left[\begin{array}{c} I\\ -I \end{array}\right]$, $a=\left[\begin{array}{c} -\bar{x}\\ \underline{x} \end{array}\right]$, $B=\left[\begin{array}{c} I\\ -M_{11}^{-1}M_{12} \end{array}\right]$, $b=-\left[\begin{array}{c} {\ddot{q}}_{a\_fl}^{in}\\ M_{11}^{-1}\left(V_{1}+G_{1}\right)+{\ddot{q}}_{u\_fl}^{in} \end{array}\right]$. Furthermore, w is the corresponding weight, and $\underline{x}$ and $\bar{x}$ are the upper and lower boundaries of the joint accelerations.

## 5. Simulation and Results

The proposed controller was implemented on MATLAB/Simulink with a fixed sample cycle (4 ms) to achieve repeatable vertical jumping cycles for a planar three-link robot. In the flight phase, we want to control the robot’s hips, knees, and ankles so that they remain near the desired position, but this is an overconstrained problem. The angles of the ankle and hip are particularly important for the landing posture, so we chose large weights for ankle and hip and very small weights for the other joints. The final weight values w=diag(0.0001,0.0001,1,0.01,0.6) were used.

**Figure 7:**
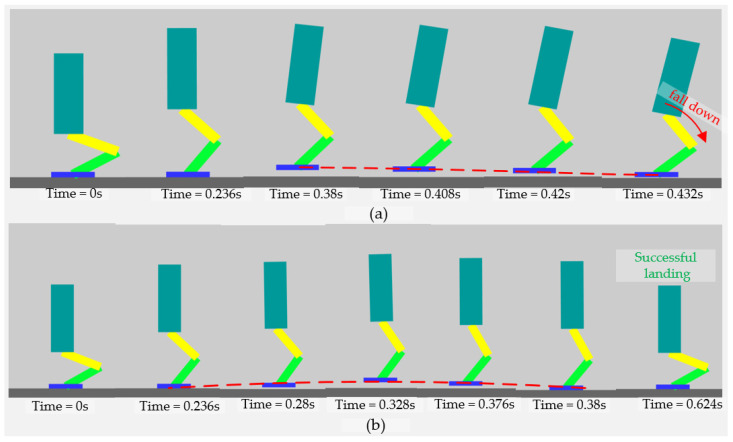
Demonstration of different jump cases with different controllers in the flight phase. (**a**) A failed jump and (**b**) a successful jump.

As depicted in Figure 7, the robot experienced both a failed landing and a successful landing. To verify the effectiveness of the flight phase controller, we chose the same controller for the two scenarios in the stance phase without controlling the angular momentum in the launch phase, which caused the angular momentum to exceed 30 N·m·s after the robot’s foot left the ground. When the robot foot left the ground, we locked the actuated joints to keep the angles of actuated joints stationary in the jump shown in Figure 7a and used the flight phase controller described in Section 4.2 for the jump shown in Figure 7b.

As shown in Figure 7a, when the actuated joints are locked, the robot rotates around the CoM because of the conservation of angular momentum, which causes the posture to deviate far from the desired value and causes the robot to fail to land.

In Figure 7b, the rotation of actuated joints compensate for the rotation of the robot caused by the conservation of angular momentum, which maintained the robot’s posture near the desired value, so the robot was able to land successfully.

**Figure 8:**
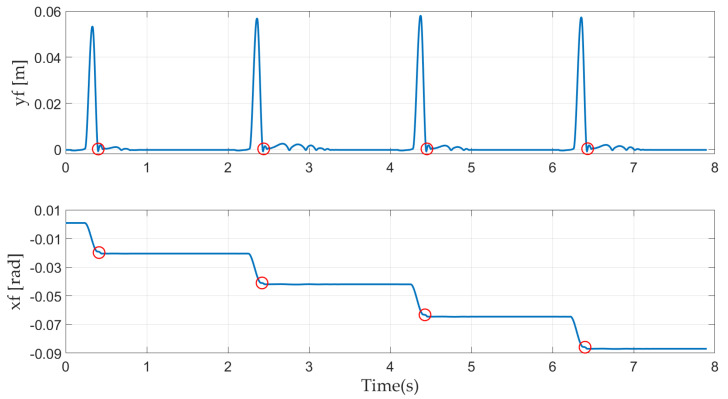
Foot trajectories in the x and y directions.

In Figure 8, foot trajectories are plotted along the x-axis (bottom) and y-axis (top). The red circles indicate the position of the foot when the robot lands. A cyclic position for the foot in both the x and y directions indicates that the robot realizes a cyclic jump. During the stance phase, the foot remains stationary on the ground except at the landing moment, and the sliding and oscillation of the robot along the x-axis and y-axis due to the impact force is small enough to be considered negligible. The foot trajectories along the x-axis in Figure 8 show that the actuated joints compensate for the rolling of the robot in the flight phase, which causes the position of the foot in the landing position to deviate periodically from the launch position.

**Figure 9:**
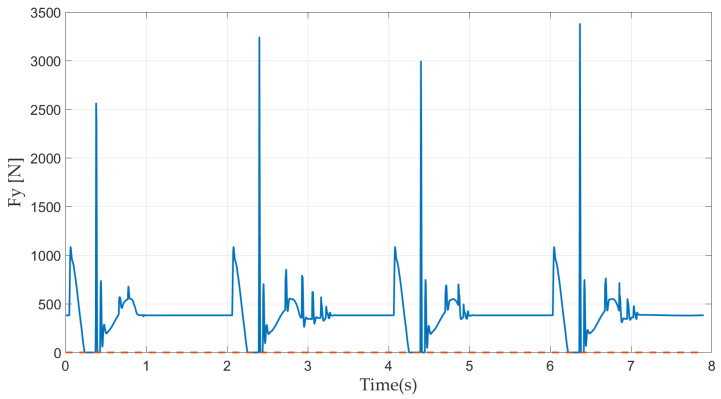
Ground reaction force.

In Figure 9, the periodically occurring zero ground reaction force indicates that the robot’s foot successfully achieves periodic flight. When the foot touches the ground after the flight phase, the touch-down impact can be observed. The touch-down impact acting on the foot is very large because of the position of the input joints.

**Figure 10:**
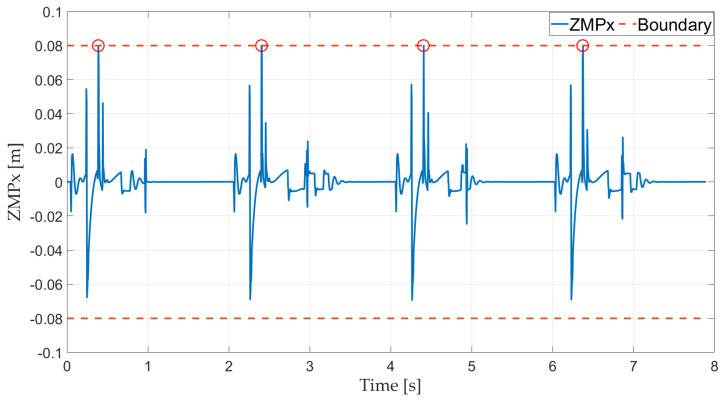
Foot ZMP in the x-axis.

Foot boundaries (red dotted line) and the ZMP along the x-axis (blue solid line) are presented in Figure 10. The results show that the ZMP response is always within the supporting polygon except at touch-down because of the touch-down impact, which reveals that the robot is stable in the stance phase and the controller for stabilizing the simplified robot model is effective.

**Figure 11:**
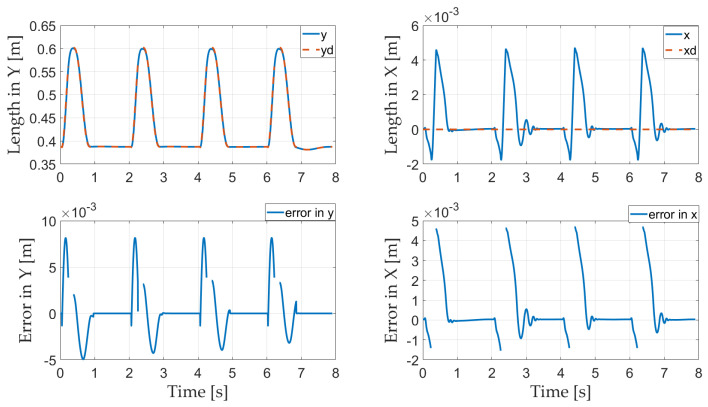
Trajectory of the CoM and its error.

The trajectory of the CoM and the corresponding error are presented in Figure 11. As the CoM is not controlled during the flight phase, the CoM error disappears during the flight phase. The trajectory of the CoM shows that the robot tracks the reference trajectories in both x and y directions well. In the stance phase, the HQP controller maintains the error of the CoM of the robot at the millimeter scale, instead of the centimeter scale as reported in [40]; the maximum errors of the CoM in the x and y directions are, respectively, 0.0047 and 0.0082 m in the present study and 0.0761 and 0.0392 m in [40]. The maximum errors in the x and y directions in [40] are thus 16.19 and 4.78 times those in the present paper, respectively, demonstrating that the HQP controller ensures strict prioritization in the overconstrained control problem.

**Figure 12:**
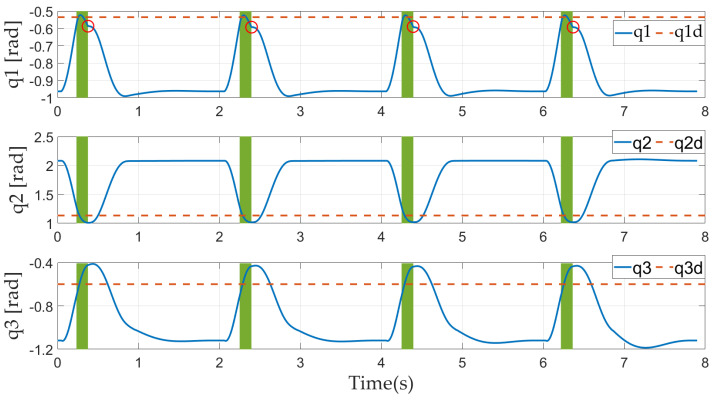
Joint trajectories in simulation.

Figure 12 displays the joint trajectories (blue solid lines), reference values in the flight phase (red dotted lines), and touch-down times (solid red circles).

In the stance phase, the joint trajectories are all within the joint limitations and smooth except at the touch-down time owing to the touch-down impact. Due to the greater impact acting on the ankle joint, the velocity of the ankle joint changes abruptly. The knee and hip joints are less affected, and the changes in velocities can be negligible.

During the flight phase, the controller is able to successfully restrict the robot’s posture to be near the desired constant value to prepare for a successful landing. The larger weight of the ankle joint means that the deviations from the desired angle for the ankle are much less than those for the knee and hip.

## 6. Discussion

Previous studies have adopted various approaches to realize a robot’s jumping motion. In overconstrained tasks for robots, adding different weights to the control tasks can only represent relative priority and it is difficult to guarantee strict prioritization; pseudo-inverse-based hierarchical methods cannot deal with the inequality constraints. In the flight phase, none of these methods allow the controller to keep the position and attitude of the robot near the desired values.

We proposed a method that incorporates the vertical jumping problem of the robot into a QP-based framework. In the stance phase, the HQP controller for a degenerative mass point model is used to consider the constraints and guarantee strict prioritization. During the flight phase, the split dynamic equation is unified into a QP controller to keep the robot’s posture near a desired position.

In the stance phase, the maximum errors of the CoM are, respectively, 0.0047 and 0.0082 m in the x direction and y direction, whereas they are 0.0761 and 0.0392 m in [40]. In [40], the priorities of tasks are expressed by adding different weights to different tasks, which only guarantees relative priorities and not strict prioritization, and the CoM tracking task cannot be accurately executed and is coupled with other less important tasks, reducing the accuracy of the CoM task. Therefore, the maximum errors in the x direction and y direction in [40] are 16.19 and 4.78 times those in this paper, respectively.

In the flight phase, the rotations of actuated joints compensate for the rotation of the robot due to the conservation of angular momentum, which maintains the robot’s posture near the desired value, so the robot lands successfully.

We validated the effectiveness of the controller by comparing simulation results.

## 7. Conclusions

This paper proposed an online optimal control method that incorporates the vertical jumping problem of a robot into a QP-based framework. During the stance phase, the HQP controller for a degenerative mass point model is introduced to consider constraints and guarantee strict prioritization in the overconstraint tasks. In the flight phase, the split dynamic equation is unified into a QP controller to keep the robot’s posture near a desired position.

Through simulation, we demonstrated the QP-based framework. First, the HQP controller in the stance phase reduces the maximum errors of the CoM in the x direction and y direction by factors of 16.19 and 4.78, respectively, compared with previous work, which demonstrates that the HQP controller can accurately execute the CoM tracking task and guarantee strict CoM prioritization. Second, the use of different controllers in the flight phase leads to different landing cases, which validates the use of the QP controller to maintain the robot’s posture near a desired constant value in the flight phase.

In future work, we will extend this method to a 3D case for the full dynamic model, in which sagittal and lateral motion can occur. To this end, a substantial amount of work must be carried out; for instance, the control of the degenerative model in the jumping motion should be extended to the control of a full dynamics model. Owing to the compatibility and scalability of the online unified framework based on QP, the extension of the 3D jump control problem can be successfully incorporated into this unified framework.

## Figures and Tables

**Table 1:** Inertial robot parameters used in the simulation.

Link (i)	$m_{i}\;(kg)$	$I_{ci}\;(kg\times m^{2})$	$L_{i}\;(m)$	$L_{ci}\;(m)$
Shank (1)	5.04	0.0718	0.3	0.15
Thigh (2)	14.26	0.396	0.3	0.15
Torso (3)	16.38	0.8169	0.6	0.2186

**Table 2:** Parameter vector and weights in the optimization for trajectory generation.

$\underline{q}$	$\dot{\underline{q}}$	$\ddot{\underline{q}}$	$w_{\tau }$	$w_{\tau ch}$
[−1.14, 0, −1.57 ]	[−160, −360, −200]	−[360, 560, 500]	Diag ([0.6, 0.6, 0.6])	Diag ([0.0006, 0.0006, 0.0006])
$\bar{q}$	$\bar{\dot{q}}$	$\bar{\ddot{q}}$	$w_{q}$	$w_{qch}$
[0, 2.28, 0]	[300, 300, 300]	[600, 600, 600]	Diag ([1, 1, 1])	Diag ([0.001, 0.001, 0.001])

**Table 3:** Vectors in the optimization of trajectory planning.

Priority	Description	$B_{i}$	$b_{i}$
1	CoM	$\left[\begin{array}{c} J_{x}\\ J_{y} \end{array}\right]$	$\left[\begin{array}{c} {\dot{J}}_{x}{\dot{q}}_{s}-{\ddot{x}}_{CoM}^{in}\\ {\dot{J}}_{y}{\dot{q}}_{s}-{\ddot{y}}_{CoM}^{in} \end{array}\right]$
2	Joints, changes	$\left[\begin{array}{c} I\\ I \end{array}\right]$	$\left[\begin{array}{c} {\ddot{q}}_{s}^{in}\\ {\ddot{q}}_{s}^{N} \end{array}\right]$

## Data Availability

Data sharing not applicable.

## References

[1] Blickhan R. (1989). The Spring-Mass Model for Running and Hopping. J. Biomech..

[2] Vermeulen J., Lefeber D., Verrelst B. (2003). Control of Foot Placement, Forward Velocity and Body Orientation of a One-Legged Hopping Robot. Robotica.

[3] Ugurlu B., Kawamura A. Real-Time Jumping Trajectory Generation for a One Legged Jumping Robot. Proceedings of the 2008 34th Annual Conference of IEEE Industrial Electronics.

[4] Ugurlu B., Kawamura A. Eulerian ZMP Resolution: Real-Time Jogging and Jumping Trajectory Planning for Bipedal Robots. Proceedings of the 2009 IEEE/ASME International Conference on Advanced Intelligent Mechatronics.

[5] Yim J.K., Singh B.R.P., Wang E.K., Featherstone R., Fearing R.S. (2020). Precision Robotic Leaping and Landing Using Stance-Phase Balance. IEEE Robot. Autom. Lett..

[6] Hodgins J.K., Raibert M.N. (1991). Adjusting Step Length for Rough Terrain Locomotion. IEEE Trans. Robot. Automat..

[7] Ugurlu B. (2010). ZMP-Based Online Jumping Pattern Generation for a One-Legged Robot. IEEE Trans. Ind. Electron..

[8] Caron S., Kheddar A., Tempier O. Stair Climbing Stabilization of the HRP-4 Humanoid Robot Using Whole-Body Admittance Control. Proceedings of the 2019 International Conference on Robotics and Automation (ICRA).

[9] Ott C., Roa M.A., Hirzinger G. Posture and Balance Control for Biped Robots Based on Contact Force Optimization. Proceedings of the 2011 11th IEEE-RAS International Conference on Humanoid Robots.

[10] Feng S., Whitman E., Xinjilefu X., Atkeson C.G. (2015). Optimization-Based Full Body Control for the DARPA Robotics Challenge: Optimization-Based Full Body Control For The DARPA Robotics Challenge. J. Field Robot..

[11] Lee S.-H., Goswami A. Ground Reaction Force Control at Each Foot: A Momentum-Based Humanoid Balance Controller for Non-Level and Non-Stationary Ground. Proceedings of the 2010 IEEE/RSJ International Conference on Intelligent Robots and Systems.

[12] Jiang X., Chen X., Yu Z., Zhang W., Meng L., Huang Q. (2018). Motion Planning for Bipedal Robot to Perform Jump Maneuver. Appl. Sci..

[13] Ahn D., Cho B.-K. Optimal Periodic Hopping Trajectory Generation for Legged Robots. Proceedings of the 2018 IEEE/ASME International Conference on Advanced Intelligent Mechatronics (AIM).

[14] Ahn D., Cho B.-K. (2020). Optimal Standing Jump Trajectory Generation for Biped Robots. Int. J. Precis. Eng. Manuf..

[15] Wan X., Urakubo T., Tada Y. (2015). Optimization of Jumping Motion of a Legged Robot for Different Take-off Postures. J. Mech. Sci. Technol..

[16] Nguyen Q., Powell M.J., Katz B., Carlo J.D., Kim S. Optimized Jumping on the MIT Cheetah 3 Robot. Proceedings of the 2019 International Conference on Robotics and Automation (ICRA).

[17] Ding Y., Park H.-W. Design and Experimental Implementation of a Quasi-Direct-Drive Leg for Optimized Jumping. Proceedings of the 2017 IEEE/RSJ International Conference on Intelligent Robots and Systems (IROS).

[18] Sentis L., Khatib O. (2005). Synthesis Of Whole-Body Behaviors Through Hierarchical Control Of Behavioral Primitives. Int. J. Human. Robot..

[19] Nakamura Y., Hanafusa H., Yoshikawa T. (1987). Task-Priority Based Redundancy Control of Robot Manipulators. Int. J. Robot. Res..

[20] Kim D., Di Carlo J., Katz B., Bledt G., Kim S. (2019). Highly Dynamic Quadruped Locomotion via Whole-Body Impulse Control and Model Predictive Control. arXiv.

[21] Hutter M., Sommer H., Gehring C., Hoepflinger M., Bloesch M., Siegwart R. (2014). Quadrupedal Locomotion Using Hierarchical Operational Space Control. Int. J. Robot. Res..

[22] Herzog A., Righetti L., Grimminger F., Pastor P., Schaal S. Balancing Experiments on a Torque-Controlled Humanoid with Hierarchical Inverse Dynamics. Proceedings of the 2014 IEEE/RSJ International Conference on Intelligent Robots and Systems.

[23] Herzog A., Rotella N., Mason S., Grimminger F., Schaal S., Righetti L. (2016). Momentum Control with Hierarchical Inverse Dynamics on a Torque-Controlled Humanoid. Auton. Robot..

[24] Kanoun O., Lamiraux F., Wieber P.-B. (2011). Kinematic Control of Redundant Manipulators: Generalizing the Task-Priority Framework to Inequality Task. IEEE Trans. Robot..

[25] de Lasa M., Mordatch I., Hertzmann A. (2010). Feature-Based Locomotion Controllers. ACM Trans. Graph..

[26] Aoustin Y., Formalskii A.M. (2013). Modeling, Control and Simulation of Upward Jump of a Biped. Multibody Syst. Dyn..

[27] Aoustin Y., Formalskii A. (2013). Upward Jump Of A Biped. Int. J. Human. Robot..

[28] Xiong X., Ames A.D. Bipedal Hopping: Reduced-Order Model Embedding via Optimization-Based Control. Proceedings of the 2018 IEEE/RSJ International Conference on Intelligent Robots and Systems (IROS).

[29] Tamaddoni S.H., Jafari F., Meghdari A., Sohrabpour S. (2010). Biped hopping control bazsed on spring loaded inverted pendulum model. Int. J. Human. Robot..

[30] Kuindersma S., Deits R., Fallon M., Valenzuela A., Dai H., Permenter F., Koolen T., Marion P., Tedrake R. (2016). Optimization-Based Locomotion Planning, Estimation, and Control Design for the Atlas Humanoid Robot. Auton. Robot..

[31] Miyadaira A.N., Kolm Madrid M., Garcia J.C., Gualda D., Delai A.L. (2018). Squat Vertical Jump of a 3DOF Robot Leg over an Inclined Plane: Analysis with Joint Torque Profile Approximation. IEEE Latin. Am. Trans..

[32] Schlossman R., Thomas G.C., Campbell O., Sentis L. (2018). Exploiting the Natural Dynamics of Series Elastic Robots by Actuator-Centered Sequential Linear Programming. arXiv.

[33] Zhang J., Song G., Li Y., Qiao G., Song A., Wang A. (2013). A Bio-Inspired Jumping Robot: Modeling, Simulation, Design, and Experimental Results. Mechatronics.

[34] Nunez V., Drakunov S., Nadjar-Gauthier N., Cadiou J.C. Control Strategy for Planar Vertical Jump. Proceedings of the 12th International Conference on Advanced Robotics.

[35] Nunez V., Nadjar-Gauthier N. Control Strategy for Vertical Jump of Humanoid Robots. Proceedings of the 2005 IEEE/RSJ International Conference on Intelligent Robots and Systems.

[36] Park H.-W., Park S., Kim S. Variable-Speed Quadrupedal Bounding Using Impulse Planning: Untethered High-Speed 3D Running of MIT Cheetah 2. Proceedings of the 2015 IEEE International Conference on Robotics and Automation (ICRA).

[37] Astley R.J., Stol K.A., Harrington J.J. (1998). Modelling the Elastic Properties of Softwood.

[38] Hudson P.E., Corr S.A., Wilson A.M. (2012). High Speed Galloping in the Cheetah (Acinonyx Jubatus) and the Racing Greyhound (Canis Familiaris): Spatio-Temporal and Kinetic Characteristics. J. Exp. Biol..

[39] Haberland M. The Effect of Swing Leg Retraction on Running Energy Efficiency. Proceedings of the 2011 IEEE/RSJ International Conference on Intelligent Robots and Systems.

[40] Tian D., Gao J., Liu C., Shi X. (2021). Simulation of Upward Jump Control for One-Legged Robot Based on QP Optimization. Sensors.

